# Empathy Unmasked: Patient Perception of Physician Empathy in an Oncologic Emergency Setting. A Randomized Controlled Trial Comparing Personal Protective Equipment Wear versus Unmasked Video Communication

**DOI:** 10.1016/j.jemermed.2024.08.005

**Published:** 2024-08-13

**Authors:** Kathryn J. Burk, John J. Stroh, Kelly Larkin, Patrick Chaftari, Denise M. Langabeer, Jazmin R. Menendez, Jeanie F. Woodruff, Jan Hargrave, Hongxu Zhu, James P. Long, Sai-Ching J. Yeung, Kumar Alagappan, Aiham Qdaisat

**Affiliations:** * Department of Emergency Medicine, The University of Texas MD Anderson Cancer Center, Houston, Texas; † Cancer Prevention Research Training Program, The University of Texas MD Anderson Cancer Center, Houston, Texas; ‡ Department of Biostatistics, The University of Texas MD Anderson Cancer Center, Houston, Texas

**Keywords:** emergency, telemedicine, telehealth, oncology, cancer, personal protective equipment, virtual, patient satisfaction, empathy, relationship

## Abstract

**Background:**

Amidst the COVID-19 pandemic, telemedicine emerged as an important option that supports and facilitates clinical practice, however, its usefulness in emergency settings that treat patients with cancer is unclear.

**Objective:**

To evaluate patient perception of physician empathy in an emergency oncology setting, comparing video interaction to an in-person with personal protective equipment (PPE) approach.

**Methods:**

In this single-center, prospective, cross-sectional, survey-based randomized controlled trial, patients were randomized 1:1 for the concluding conversation done in-person which included either interacting with physicians wearing PPE or video interaction with physicians without PPE (virtual). Patients’ perceptions of the physicians’ relational empathy were assessed and compared for each group by using the Consultation and Relational Empathy (CARE) Measure and the Perception of Physician Compassion measure.

**Results:**

Patients (n = 106) in both the PPE and virtual arms provided favorable responses to all questions. The mean overall CARE scores for the PPE and virtual arms were 45.02 and 44.43, respectively (difference, 0.58 [95% CI: −2.10, 3.30]). Regarding the linear physician compassion scores, patients in the virtual arm appeared to consider physicians to be warmer (difference, −0.42 [95% CI: −0.87, 0.04]) but less pleasant (difference, 0.33 [95% CI: −0.40, 1.10]) than did patients in the PPE arm.

**Conclusions:**

Cancer patients presenting to the emergency department perceive empathy and compassion equally when approached by physicians virtually without PPE or in person while wearing PPE. Virtual services for specific aspects of clinical practice during emergency department visits in an oncology setting can be implemented to ensure safer interactions between patients and physicians without compromising the physician-patient relationship.

## Introduction

The COVID-19 pandemic elevated telemedicine into a mainstream modality for communications in health care (1). During the COVID-19 pandemic, telemedicine evolved as one option that supports particular fields within the clinical practice including specific services within the oncology practice (2,3). According to the American Hospital Association, the adoption of telemedicine services in U.S. hospitals, indicated by full or partial implementation of computerized telehealth systems had grown from 35% in 2010 to 76% in 2017 (4), and a more recent study revealed that the pandemic further increased the demand for telemedicine services to bridge access to care (5). Telemedicine can be used to monitor, diagnose, treat disease, and counsel patients when in-person care is not feasible. Additionally, it may lend to other benefits including convenience and cost savings (6).

Even though facial coverings have been widely embraced by the general public during the COVID-19 pandemic, the use of masks and other personal protective equipment (PPE) in clinical settings may also adversely impact one’s situational awareness (e.g., ability to perceive and understand one’s situation), well-being as well as the resilience of both patients and clinicians within a healthcare delivery system (7,8). The practice of medicine is about human connection—a connection between healers and the sick (9). Empathy is vital to this connection, as it demonstrates that the healthcare practitioner understands the patient’s condition, perspective, feelings, along with the ability to communicate in a manner to achieve understanding, validate the accuracy of the information given, and act on that understanding in a helpful, therapeutic manner (10). Empathy and interpersonal connection can also be expressed nonverbally, such as through body language and facial expressions. Such nonverbal communication strengthens the therapeutic alliance between patient and healthcare practitioner and influences patient satisfaction, trust, and adherence to care plans, thereby bolstering clinical outcomes (11,12).

As opportunities for telemedicine have increased in a variety of clinical settings, the effectiveness of managing patients with complicated diseases, such as cancer, remains unclear. Studies suggest that patients diagnosed with cancer have shown to have conflicting views on the use of telemedicine, primarily because the use of personal protective equipment hinders healthcare providers’ ability to fully interact with patients through facial expressions and voice (13,14). This was also evident in a primary care randomized, controlled study in which over 1000 patients felt interpersonal communication and empathy were perceived negatively when the physician presented with a face mask during consultations (15). Results seem to suggest that patients preferred to see their physician’s face when discussing their healthcare ailments.

Although several reports have described patterns of telemedicine use in the emergency department (ED) setting, challenges exist—especially because patients generally present with urgent or emergency concerns requiring the physician to assess the patient’s history, perform physical examination, and order ancillary services such as laboratory and imaging studies. In addition, cancer patients often have a significant symptom burden resulting from both their disease and associated therapies which subsequently, requires a more meticulous assessment.

To assess the perception of physician empathy either in person with PPE or by video interaction, this prospective study described herein evaluated and compared how cancer patients in an ED setting preferred to communicate with their physician.

## Methods

### Study Recruitment, Setting, and Randomization

This single-center, prospective, cross-sectional, survey-based randomized controlled trial was performed under a protocol approved by the Institutional Review Board of our institution (2020-1298; ClinicalTrials.gov identifier: NCT05102656). The sample size was calculated by using the assumption that, to reach a power of 90% at a significance level of 0.05, the study should include 53 participants in each arm to detect a five-point difference in the means of the CARE Measure score, considering a standard deviation of 7.84. This assumption was based on a similar study (15) that used the CARE measure to assess the effect of face masks on physician empathy and relational continuity.

All patients for this study presented to the ED from June 17, 2021, to April 31, 2022. This ED is a specialized unit within a comprehensive cancer center that focuses on the treatment and management of emergent/urgent medical conditions related to cancer and its treatment. Nearly, 95% of all patients treated in our ED are diagnosed with cancer (16). A small percentage are patients with benign conditions, visitors, or employees.

Each patient was triaged and roomed as per usual procedures. Patients sent directly for hospital admission or did not require additional workup or a second conversation for communication of the results were excluded. Other exclusion criteria were taken into consideration if a physician observed that the patient was too ill to participate. The inclusion criteria were 1) at least 18 years of age, 2) able to speak and write in English, 3) able to understand and willing to sign a written informed consent document, and 4) willing and able to complete survey assessments.

A table for randomizing patients was generated at the beginning of the study by using permuted block randomization with a random block constellation that randomly assigned each patient to a 1:1 ratio of the two study arms (17,18).

All patients were seen by an emergency physician in person with PPE for the initial history and physical examinations inclusive of an explanation of the proposed workup. After the physician leaves the room, the patient is then approached by a research assistant about participating in the study. If consent to participate is received, the patient is then randomized to either receive their test results (e.g., laboratory, imaging study, or other test results) in an in-person follow-up with the physician wearing PPE (PPE arm) or by a video call with a physician not wearing Virtual (virtual arm). The treating physicians were unblinded and were notified of their patient’s study arm only after the initial conversation with the patient. If randomized to the virtual arm, the patient was provided with a tablet or portable computer for all subsequent conversations (beyond the initial history and physical examinations). A research assistant was available for technical support if needed. The treating physician on the Virtual arm was visible from the waist up, and their hand gestures also were visible via the tablet or portable computer. If randomized to the PPE arm, further conversations with the treating physician were conducted in person, with the provider wearing PPE in the same manner as in the initial examination.

### Data Collection and Patient Survey

Demographic data were collected from the patients’ electronic medical records. Patients’ perceptions of their healthcare practitioners’ relational empathy were assessed using surveys. All study assessments were completed in the privacy of the patient’s room before the final disposition from the ED, either electronically or by using a paper version of the survey. Patients were asked to complete two assessments: 1) the Consultation and Relational Empathy (CARE) Measure and 2) the Perception of Physician Compassion linear measure.

The CARE Measure is a validated patient-reported questionnaire that avoids any attempt to judge the quality of technical care (such as the quality of a clinical examination); rather, it is based on a broad definition of empathy within the context of a therapeutic relationship during a consultation (10,19,20). The phrasing of the measurement items aims to create a holistic, patient-centered measure which holds significance for all patients regardless of their social class (19,20). The CARE Measure consists of 10 items, each of which is scored on a scale of 1 to 5: 1 = poor, 2 = fair, 3 = good, 4 = very good, and 5 = excellent. The scores for all 10 items are added, resulting in a maximum possible score of 50 and a minimum possible score of 10. As the CARE measure offers a foundation grounded in theory and rigorous psychometric standards for evaluating physicians’ empathy, different quantitative and qualitative studies examined and utilized both the CARE measure individual items/subscales and the CARE measure total score (21–25). The CARE measure items provide a unidimensional assessment of physician empathy in the affective and cognitive domains, and hence, the importance of the responses to the individual items along with the total score (22,23). For this study, up to two “Not Applicable” responses or missing values were allowed and were replaced with the average score for the remaining items. Questionnaires with more than two missing values or “Not Applicable” responses were removed from the analysis.

Patients rated the physician’s compassion by using a semantic differential scale containing five pairs of physician characteristics: warm/cold, pleasant/unpleasant, compassionate/distant, sensitive/insensitive, and caring/uncaring (26). Each paired physician characteristic was presented on a 10-cm line on which the negative characteristic for each pair (cold, unpleasant, distant, insensitive, and uncaring) was placed on the left of the line, and the positive characteristic (warm, pleasant, compassionate, sensitive, caring) was placed on the right of the line. The patient was instructed to rate the healthcare practitioner by putting an X on the line for each relevant paired characteristic. The distance of the X from the left anchor was then calculated in centimeters, such that each item had a possible range of 0 to 10 points. The five items were summed, reflecting the extent to which the patient perceived the physician as compassionate.

An additional evaluation was performed to examine the patients’ generation group. The generational categories groups were defined as Traditionalists (born before 1945), Baby Boomers (born 1946–1964), Generation X (born 1965–1980), Generation Y/Millennials (born 1981–1995), and Generation Z (1996–2010) (27,28).

### Statistical Analysis

Descriptive statistics, including number and percentage, mean and standard deviation (SD), median and interquartile range (IQR), were used to describe and compare patient characteristics. Significance was appraised by using a chi-square test, the Fisher exact test, the Student’s *t*-test, or nonparametric tests (Wilcoxon-Mann-Whitney test) when the normality assumption was not met. Two-way analysis of variance was used to determine whether physicians performed better in videoconferences or in-person as well as to assess any generational differences in patient perception of healthcare practitioner empathy within the two delivery settings. Physicians who treated <10 patients were grouped into one group “Others”. The *p* values less than 0.05 (two-tailed test) were considered significant. All statistical analyses were performed using R software (version 4.1.2, The R Foundation, http://www.r-project.org).

## Results

Of 216 patients assessed for study eligibility, 107 patients were enrolled and randomly allocated to either the PPE arm or the virtual arm (Figure 1). Of the enrolled patients, 106 who completed the survey (53 per arm) are included in the final analysis. Reasons for not participating included: preferring in-person/face-to-face interaction (n = 67; 61.5%); not being interested in the study (n = 38; 34.9%); or other reasons (n = 4; 3.7%).

Table 1 summarizes the main sociodemographic characteristics of the patients by study arm. The median ages in the PPE and virtual arms were 64 years (IQR, 55–71 years) and 58 years (IQR, 47–67 years), respectively. Most of the patients were White (75%) and not Hispanic or Latino (88%). Generationally, most patients were either Baby Boomers (47%) or Generation X (27%). Given the age inclusion criterion (≥18 years), the Generation Z group had only one patient. The characteristics of the treating physicians are summarized in Table S1. The median age of the eight treating physicians involved in this study was 59 years (IQR, 55-63 years), with three (37.5%) being female and seven (87.5%) having an M.D. as the level of training. The median years of post-graduation was 26 years (IQR, 23–29 years).

With regard to the CARE Measure, patients in both arms provided favorable responses to all questions (Table 2). The mean score for all individual CARE components was >4.5. The largest score difference between the two arms (0.14) was in the story (i.e., “letting you tell your ‘story’”) component of the CARE Measure. No score difference for the “showing care and compassion” component of the CARE Measure was observed. The mean overall CARE scores did not differ significantly between the PPE and virtual arms, (45.02 and 44.43, respectively; difference, 0.58 [95% CI: −2.10, 3.30]).

In the linear empathy measure, patients in the virtual arm appeared to consider physicians to be warmer (difference, −0.42 [95% CI: −0.87, 0.04]) but less pleasant (difference, 0.33 [95% CI: −0.40, 1.10]) than did patients in the PPE arm (Table 3). However, these differences were not significant. The scores for the compassionate, caring, and sensitive components in the two arms were comparable.

The results of the two-way analysis of variance by study arm, physician, and study arm-physician interaction terms indicated that the physician was significantly associated with the total CARE score (*p* < 0.001) but that neither the study arm nor the study arm-physician interaction was significantly associated with the total score (Figure 2 and Table S2).

In a two-way analysis of variance with study arm, generation, and study arm-generation interaction terms, none of these three terms were significant (Figure 3 and Table S3).

When examining the CARE Measure and the linear empathy measure stratified by different patient characteristics, there was only a significant difference in the “being positive” component of the CARE measure with higher mean scores reported among females when compared to males (4.83 vs. 4.60; *p* = 0.044), and in the warm item of the linear empathy score, with higher mean scores among patients < 60 years of age, compared to patients ≥ 60 years (9.38 vs. 8.91; *p* = 0.043). The remaining CARE item and overall CARE scores, and the linear empathy scores are reported in Table S4 (stratified by sex), Table S5 (stratified by race), and Table S6 (stratified by age groups).

## Discussion

Despite the widespread adoption of facial coverings by the general public during the COVID-19 pandemic, the wearing of masks and other PPE by healthcare providers in a clinical setting may have detrimental effects on situational awareness, the delivery of health care, and patient and clinician communication, well-being, and resilience (29,30). The results of this study supported that cancer patients presenting to the ED had similar ratings of their experience regarding interpersonal quality of care by emergency physicians and physician compassion, regardless of whether the physician was wearing PPE and interacting with the patient in person or was not wearing PPE and interacting by video.

In previous studies, investigators recognized that contact precautions (including patient isolation and PPE use) limit the number of healthcare practitioner interactions with patients and are associated with patient dissatisfaction with care (31). Masks obscure a substantial portion of the face, limiting the patient’s perception of nonverbal communication through facial expressions. When additional PPE is added, distinguishing one clinician from the next becomes almost impossible. Masks also diminish the volume and clarity of the spoken word (32). A study conducted before the pandemic found that these limitations can create a psychological barrier to the development of therapeutic relationships and trust between patient and practitioner (33). Also, previous studies showed that PPE use contributes to a heightened sense of isolation and fear for both patients and clinicians. As a result, some healthcare practitioners have resorted to pinning photographs of themselves to the front of their PPE in an attempt to bridge the divide between themselves and their patients (34). In contrast, this study demonstrated that patients perceived physician empathy and compassion equally, whether the physicians interacted with them in person while wearing PPE or via videoconference without PPE.

Telemedicine may enhance the quality of prehospital emergency medical services by improving remote consultations, helping to dispatch urgent patient transfers, and enhancing supervision by health care providers. In the ED and intensive care unit specifically, the application of telemedicine can 1) conserve PPE, 2) facilitate patient assessment and remote monitoring, 3) help specialists interact with emergency physicians or patients who are experiencing emergency conditions, 4) facilitate remote supervision of trainees, and 5) enable remote contact between the patient and family members (i.e., virtual visits) (6).

Understanding the patient experience, whether staff are wearing PPE or are unmasked, is an integral part of adapting clinical care to evolving clinical practice. An added challenge is the effect of PPE on communications between healthcare providers, the patient, and the patient’s family (14). Physician willingness to use telemedicine even after the COVID-19 pandemic is very high, with reasons that include safety for both the patient and the physician and optimizing medical resources (35,36). Thus, emergency physicians in oncologic settings may continue wearing masks and other PPE even when it is not required, supporting the safety of both the patients and the healthcare providers themselves. A video conference platform that can be integrated into certain aspects of an oncologic urgent-care setting (so long as direct interaction is not needed) has an advantage over an in-person interaction with healthcare providers wearing facial masks or full PPE, in that being able to see and read facial expressions provides real-time feedback for both patients and care providers.

It was observed in this study that patients rated physician warmth higher when the physicians and patients could see each other’s facial expressions via video interactions than when they could not in person. In contrast, patients rated physicians as more pleasant while in the room with them. Greater visibility of body language in videoconferences than in person and the physical proximity that was only possible in person may have contributed to these two differences, respectively, in physician interaction ratings. Although it was determined that physicians’ interactions with patients can be effective whether in person or virtual, regardless of the patient’s age group or sex, further research is warranted to determine the effectiveness of integrating video communication to provide healthcare for cancer patients in specific areas of the emergency setting without compromising clinical care or physician-patient relationships.

Whereas telemedicine has its merits, physicians should be aware that some patients are averse to the virtual platform. It was noted that almost half of those eligible to participate in this study declined to do so, with most declining because they preferred in-person/face-to-face interaction (61.5%). Future studies could examine other reasons that patients might want a physician to return to the patient’s room or that in-person interaction might be preferable. Willingness to use a virtual platform may increase in the routine hospital setting and decrease in cases of more critical illness. Individual patient preferences for virtual interaction should be considered. In addition, ED designers should consider the infection control precautions implemented during the COVID-19 pandemic and the utility of telemedicine in checking on patients, monitoring response to treatment, and communicating test results.

### Limitations

There are several study limitations. First, patients enrolled in this study are treated within a specialized ED at a comprehensive cancer center, which can limit the generalizability to non-specialty hospital EDs. Also, serious illness may have affected some patients’ willingness to participate. The overall results, which indicated no difference in CARE measure scores between the virtual and PPE arms and favorable responses for all linear empathy scores questions in both arms, may reflect the historically positive patient experience in the delivery of patient care by our emergency physicians or in our cancer hospital in general. Second, approximately two-thirds (67/109) of the patients who declined to participate in our study indicated “prefers in-person/face-to-face interaction” as the reason for the decline. This may have created sampling exclusion bias, in which the enrolled patients were more open to virtual interaction compared with the excluded ones. Third, although the physicians were blinded to the study arm until the second interaction, they ultimately were aware of their patient’s study arm. The Hawthorne effect could have occurred, as it is possible that knowing the patient’s study arm could have influenced the physician’s behavior and communication style, thereby blunting the in-person versus video interaction effect. Fourth, different patient baseline characteristics could have affected the outcomes of our study. Further studies are needed to better understand the association between individual patient’s baseline characteristics and their perception of physician empathy. Fifth, this study only assessed the patient’s perception of physician empathy but not the patient’s satisfaction. As these two are different, further studies are needed to assess the effect of using PPE while in-person versus unmasked video communication on the patient’s satisfaction when visiting an ED. Lastly, as the COVID-19 pandemic was an unexpected event, this study measured the telemedicine experience in the context of the pandemic and did not explore telemedicine use in settings and clinical practices not associated with COVID-19. A pre-COVID-19 study might have provided an opportunity to compare different perspectives on telemedicine, as the threat of COVID-19 induced a high level of anxiety among our cancer patient population. This could have increased the level of cognitive bias in our study, regardless of whether a physician was interacting in person and wearing PPE or in a videoconference and not wearing PPE.

## Conclusions

In this randomized clinical trial, the study showed that cancer patients presenting to the ED perceive empathy and compassion equally when approached by physicians virtually without PPE or in person while wearing PPE. Wider implementation of telemedicine in the oncologic emergency setting should be considered, given patients’ equal perception of empathy for physicians who meet with them in person while masked or by video while unmasked. Further studies are needed to identify specific services during ED visits that can be implemented using a virtual approach without compromising the physician-patient relationship, promoting safer interaction and reducing costs.

## Supplementary Material

Appendix: Suppl

Supplementary material associated with this article can be found, in the online version, at doi:10.1016/j.jemermed.2024.08.005.

## Figures and Tables

**Figure 1.**
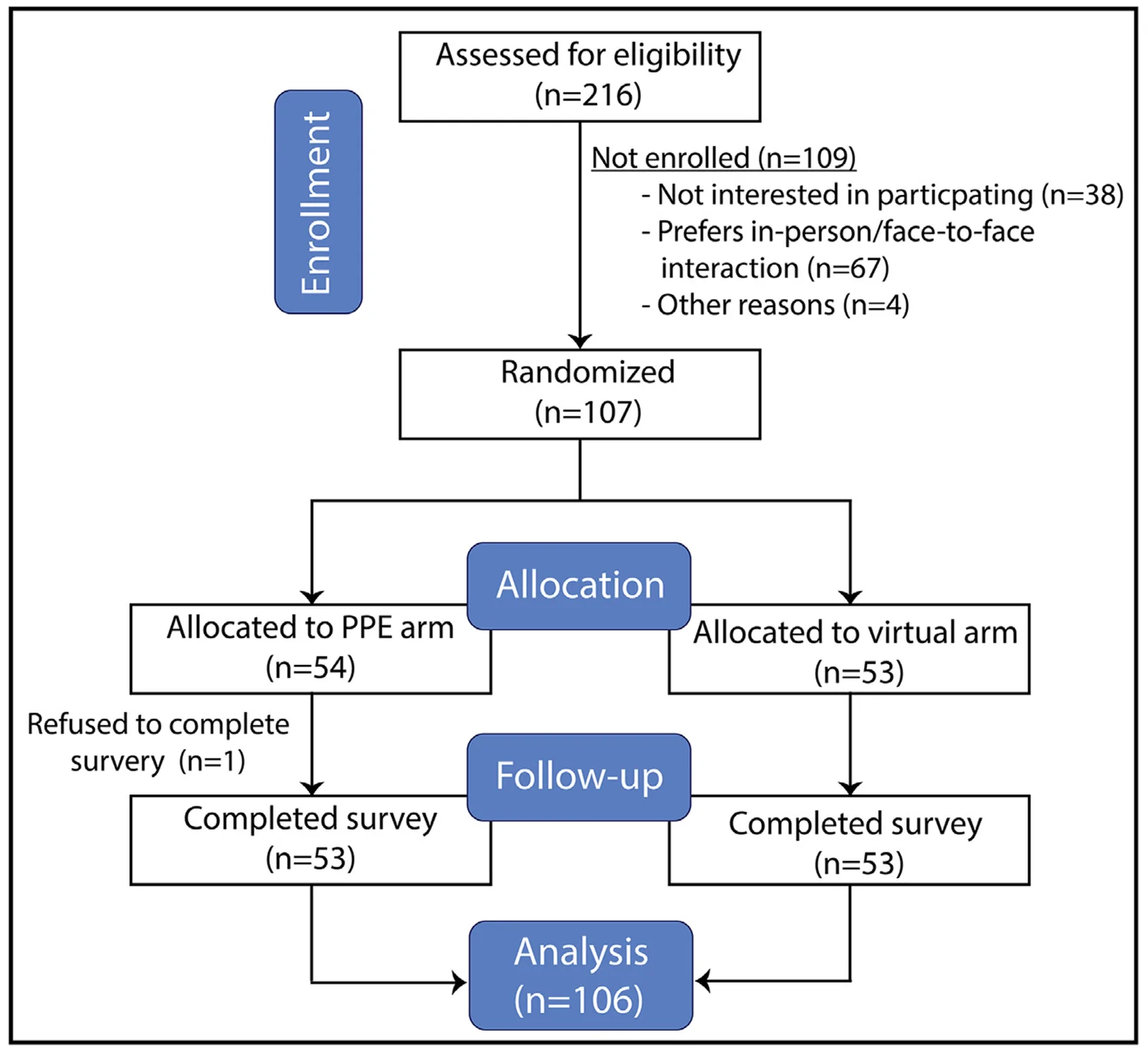
Exclusion criteria used to determine study eligibility. PPE = personal protective equipment.

**Figure 2.**
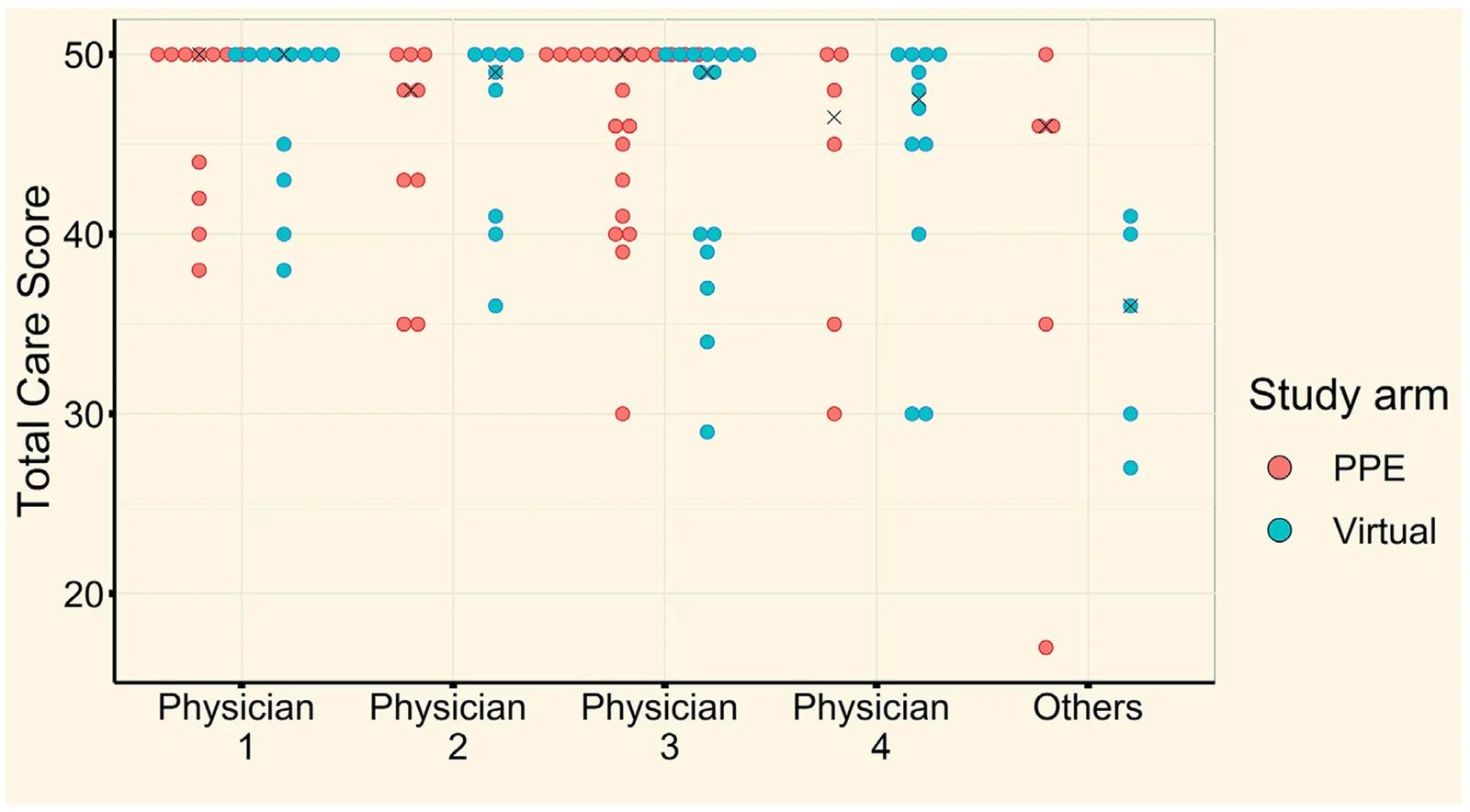
Comparison of total CARE score for each physician by study arm. Physicians who treated less than 10 patients were grouped into one group “Others.” PPE = personal protective equipment.

**Figure 3.**
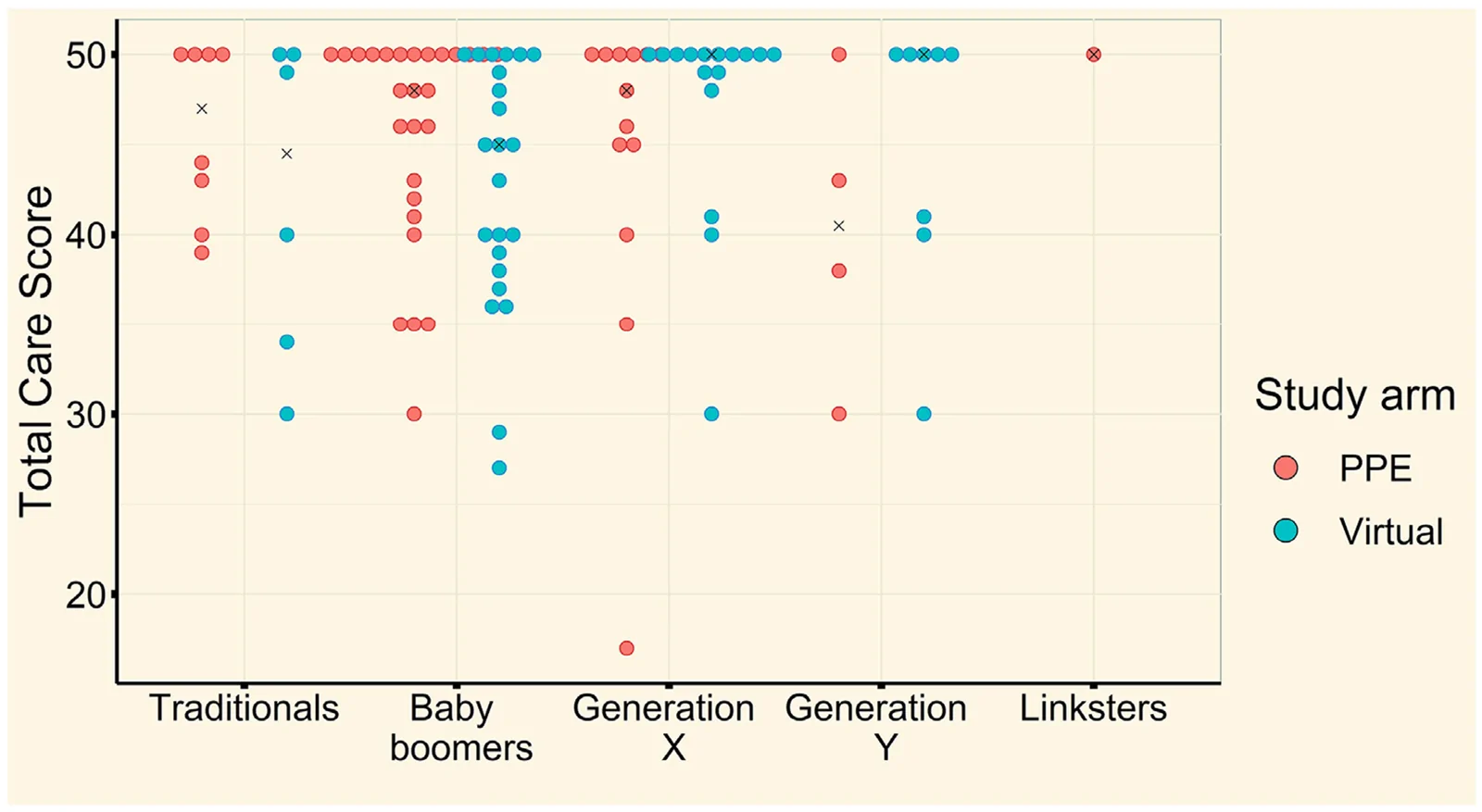
Comparison of total CARE score for each patient’s generational category group by study arm. PPE = personal protective equipment.

**Table 1.** Baseline Characteristics of the Study Cohort Stratified by Study Arm.

Characteristic	n (%)		*P*
	PPE Arm (n=53)	Virtual Arm (n=53)	
Median age, years (IQR)	64 (55–71)	58 (47–67)	0.082
Sex			0.437
Female	25 (47)	29 (55)	
Male	28 (53)	24 (45)	0.187
Race			
White	39 (74)	41 (77)	
Black	7 (13)	10 (19)	
Other	7 (13)	2 (4)	
Ethnicity			0.104
Hispanic or Latino	9 (17)	3 (6)	
Not Hispanic or Latino	43 (81)	50 (94)	
Declined to answer	1 (2)	0	
Cancer type			0.201
Brain and spinal cord	1 (1.9)	2 (3.8)	
Breast	8 (15.1)	7 (13.2)	
Colorectal	3 (5.7)	2 (3.8)	
Female genital	1 (1.9)	2 (3.8)	
Gastroesophageal	1 (1.9)	5 (9.4)	
Head and Neck	3 (5.7)	2 (3.8)	
Hepatobiliary pancreatic	4 (7.5)	6 (11.3)	
Leukemia	11 (20.8)	5 (9.4)	
Lymphoma	5 (9.4)	1 (1.9)	
Multiple Myeloma	1 (1.9)	3 (5.7)	
Prostate	5 (9.4)	2 (3.8)	
Sarcoma	5 (9.4)	2 (3.8)	
Urinary	2 (3.8)	2 (3.8)	
Others	2 (3.8)	7 (13.2)	
Non-cancer	1 (1.9)	5 (9.4)	
Generation			0.517
Traditionalists	8 (15)	6 (11)	
Baby Boomers	27 (51)	23 (43)	
Generation X	13 (25)	16 (30)	
Generation Y	4 (8)	8 (15)	
Generation Z	1 (2)	0	

Abbreviations: PPE, Personal protective equipment; IQR, interquartile range

**Table 2.** The CARE Item Scores and Overall CARE Scores by Study Arm.

Characteristic	n	Mean Score (± SD)		Difference*	95% CI
		PPE Arm	Virtual Arm		
CARE item score					
Ease	105	4.62 (0.79)	4.60 (0.63)	0.03	−0.25, 0.30
Story	103	4.65 (0.76)	4.51 (0.73)	0.14	−0.15, 0.44
Listening	104	4.58 (0.72)	4.61 (0.67)	−0.02	−0.29, 0.25
Interested	103	4.55 (0.76)	4.58 (0.61)	−0.03	−0.30, 0.24
Understanding	105	4.66 (0.71)	4.65 (0.59)	0.01	−0.25, 0.26
Compassion	104	4.67 (0.76)	4.67 (0.58)	0.00	−0.26, 0.26
Positive	102	4.75 (0.56)	4.69 (0.58)	0.06	−0.17, 0.28
Explaining	104	4.68 (0.75)	4.65 (0.59)	0.03	−0.23, 0.30
Control	91	4.60 (0.69)	4.59 (0.69)	0.01	−0.27, 0.30
Plan	104	4.56 (0.73)	4.63 (0.63)	−0.08	−0.34, 0.19
Overall CARE score	106	45.02 (6.93)	44.43 (6.96)	0.58	−2.10, 3.30

* Welch two-sample t-test. CARE item definitions: Ease, making you feel at ease; Story, letting you tell your “story”; Listening, really listening; Interested, being interested in you as a whole person; Understanding, fully understanding your concerns; Compassion, showing care and compassion; Positive, being positive; Explaining, explaining things clearly; Control, helping you to take control; Plan, making a plan of action with you.

Abbreviations: SD, standard deviation; CI, confidence interval; CARE, Consultation and Relational Empathy; PPE, personal protective equipment.

**Table 3.** Linear Empathy Scores by Study Arm.

Characteristic	N	Mean Score (± SD)		Difference*	95% CI
		PPE Arm	Virtual Arm		
Warm	106	8.92 (1.36)	9.34 (0.98)	−0.42	−0.87, 0.04
Pleasant	105	9.31 (1.39)	8.98 (2.26)	0.33	−0.40, 1.10
Compassionate	105	9.08 (1.34)	9.06 (1.89)	0.02	−0.61, 0.65
Caring	105	9.15 (1.30)	9.08 (2.02)	0.08	−0.58, 0.74
Sensitive	105	9.33 (1.10)	9.26 (1.58)	0.06	−0.46, 0.59

* Welch two-sample *t*-test.

Abbreviations: SD, standard deviation; PPE, personal protective equipment; CI, confidence interval

## Article Summary

### 1. Why is this topic important?

The physician-patient relationship is at the very center of medical practice; therefore, understanding the benefits and pitfalls of telemedicine as it relates to the physician-patient relationship is of vital importance. Given that using telemedicine in oncologic emergency care is relatively novel, the optimal balance between maintaining safety (masking and distancing) and personal interaction (facial expression and tone of voice) is unknown.

### 2. What does this study attempt to show?

This study assessed the patient perception of physician empathy in an oncologic emergency department, comparing in-person interaction with physicians wearing personal protective equipment versus video interaction with physicians not wearing personal protective equipment. The evaluation focused on the part of the visit for which the physician’s presence in the room was unessential.

### 3. What are the key findings?

During the part of their emergency department visit in which physician presence in the room was not un-needed, cancer patients perceived empathy and compassion equally when approached by physicians virtually without personal protective equipment or in person while wearing personal protective equipment. On the other hand, almost half of the patients who were approached chose not to engage with telemedicine and opted out of our study.

### 4. How is patient care impacted?

In an oncologic emergency setting, telemedicine can be useful whenever the physician’s presence in the room is unnecessary. This can promote safer interaction and reduce costs while maintaining the same level of patient care without compromising the physician-patient relationship. Further studies are needed to identify specific services that can be provided through telemedicine during emergency department visits and to increase the acceptance of such an approach among cancer patients presenting to the emergency department.
