# Supplementary material for: Empathy Unmasked: Patient Perception of Physician Empathy in an Oncologic Emergency Setting. A Randomized Controlled Trial Comparing Personal Protective Equipment Wear versus Unmasked Video Communication
Source: J Emerg Med. Author manuscript; Available in PMC 2026 Nov 4. (PMC13734283; doi:10.1016/j.jemermed.2024.08.005)
Supplement: Supplementary file 1 [file NIHMS2216205-supplement-Appendix__Suppl.docx]

**Supplemental data**

TABLES

**Table S1. Characteristics of the treating physicians**

| **Characteristic** | **n (%)** |
| --- | --- |
| Total | 8 (100) |
| Age, years, median (IQR) | 59 (55-63) |
| Sex |  |
| Female | 3 (37.5) |
| Male | 5 (62.5) |
| Years of graduation, median (IQR) | 26 (23-29) |
| Level of training |  |
| M.D. | 7 (87.5) |
| M.D., Ph.D. | 1 (12.5) |

**Table S2. Two-way analysis of variance for the association between physician, study arm, and the total Consultation and Relational Empathy (CARE) score.**

| **Source** | **Df** | **Sum of squares** | **Mean sum of squares** | **F-value** | ***P*** |
| --- | --- | --- | --- | --- | --- |
| Study arm | 1 | 9 | 9.07 | 0.210 | 0.648 |
| Physician | 4 | 779 | 194.87 | 4.505 | 0.002 |
| Study arm: physician | 4 | 82 | 20.49 | 0.474 | 0.755 |
| Residuals | 96 | 4153 | 43.26 |  |  |

**Table S3. Two-way analysis of variance for the association between patient generation, study arm, and the total Consultation and Relational Empathy (CARE) score.**

| **Source** | **Df** | **Sum of squares** | **Mean sum of squares** | **F-value** | ***P*** |
| --- | --- | --- | --- | --- | --- |
| Study arm | 1 | 9 | 9.07 | 0.189 | 0.664 |
| Generation | 4 | 100 | 24.90 | 0.520 | 0.721 |
| Study arm: generation | 3 | 268 | 89.44 | 1.867 | 0.140 |
| Residuals | 97 | 4646 | 47.90 |  |  |

**Table S4. The CARE item scores, overall CARE, and linear empathy scores by sex.**

| **Characteristic** | **Mean Score (± SD)** | | |
| --- | --- | --- | --- |
|  | **Female** | **Male** | **P** |
| CARE item score |  |  |  |
| Ease | 4.64 (0.79) | 4.58 (0.64) | 0.645 |
| Story | 4.58 (0.80) | 4.59 (0.70) | 0.939 |
| Listening | 4.67 (0.64) | 4.52 (0.74) | 0.281 |
| Interested | 4.67 (0.68) | 4.46 (0.67) | 0.127 |
| Understanding | 4.72 (0.66) | 4.60 (0.63) | 0.342 |
| Compassion | 4.74 (0.71) | 4.60 (0.64) | 0.290 |
| Positive | 4.83 (0.47) | 4.60 (0.64) | 0.044 |
| Explaining | 4.72 (0.72) | 4.61 (0.63) | 0.414 |
| Control | 4.73 (0.59) | 4.47 (0.75) | 0.070 |
| Plan | 4.69 (0.67) | 4.50 (0.67) | 0.148 |
| Overall CARE score | 45.06 (7.46) | 44.38 (6.36) | 0.620 |
| Linear empathy score |  |  |  |
| Warm | 9.31 (1.11) | 8.94 (1.26) | 0.109 |
| Pleasant | 9.00 (2.34) | 9.29 (1.26) | 0.434 |
| Compassionate | 9.23 (1.83) | 8.90 (1.40) | 0.313 |
| Caring | 9.02 (2.19) | 9.21 (0.98) | 0.563 |

CARE item definitions: Ease, making you feel at ease; Story, letting you tell your “story”; Listening, really listening; Interested, being interested in you as a whole person; Understanding, fully understanding your concerns; Compassion, showing care and compassion; Positive, being positive; Explaining, explaining things clearly; Control, helping you to take control; Plan, making a plan of action with you.

Abbreviations: SD, standard deviation; CI, confidence interval; CARE, Consultation and Relational Empathy.

**Table S5. The CARE item scores, overall CARE, and linear empathy scores by race.**

| **Characteristic** | **Mean Score (± SD)** | | |  |
| --- | --- | --- | --- | --- |
|  | **Non-White** | **White** | **P** | |
| CARE item score |  |  |  | |
| Ease | 4.54 (0.76) | 4.63 (0.70) | 0.561 | |
| Story | 4.62 (0.70) | 4.57 (0.77) | 0.797 | |
| Listening | 4.60 (0.76) | 4.59 (0.67) | 0.975 | |
| Interested | 4.44 (0.71) | 4.60 (0.67) | 0.301 | |
| Understanding | 4.62 (0.70) | 4.67 (0.63) | 0.707 | |
| Compassion | 4.64 (0.64) | 4.68 (0.69) | 0.780 | |
| Positive | 4.73 (0.60) | 4.71 (0.56) | 0.877 | |
| Explaining | 4.73 (0.60) | 4.64 (0.70) | 0.561 | |
| Control | 4.57 (0.73) | 4.60 (0.67) | 0.820 | |
| Plan | 4.65 (0.69) | 4.58 (0.67) | 0.618 | |
| Overall CARE score | 45.08 (6.50) | 44.61 (7.08) | 0.768 | |
| Linear empathy score |  |  |  | |
| Warm | 9.46 (0.76) | 9.03 (1.29) | 0.106 | |
| Pleasant | 9.36 (2.00) | 9.07 (1.85) | 0.510 | |
| Compassionate | 9.36 (2.00) | 8.97 (1.50) | 0.305 | |
| Caring | 9.16 (2.27) | 9.10 (1.49) | 0.878 | |
| Sensitive | 9.40 (2.00) | 9.26 (1.10) | 0.661 | |

CARE item definitions: Ease, making you feel at ease; Story, letting you tell your “story”; Listening, really listening; Interested, being interested in you as a whole person; Understanding, fully understanding your concerns; Compassion, showing care and compassion; Positive, being positive; Explaining, explaining things clearly; Control, helping you to take control; Plan, making a plan of action with you.

Abbreviations: SD, standard deviation; CI, confidence interval; CARE, Consultation and Relational Empathy.

**Table S6. The CARE item scores, overall CARE, and linear empathy scores by age group.**

| **Characteristic** | **Mean Score (± SD)** | | |  |
| --- | --- | --- | --- | --- |
|  | **Age <60 years** | **Age ≥ 60 years** | **P** | |
| CARE item score |  |  |  | |
| Ease | 4.66 (0.80) | 4.56 (0.63) | 0.492 | |
| Story | 4.56 (0.90) | 4.60 (0.60) | 0.801 | |
| Listening | 4.65 (0.69) | 4.55 (0.69) | 0.430 | |
| Interested | 4.67 (0.72) | 4.46 (0.64) | 0.118 | |
| Understanding | 4.72 (0.70) | 4.60 (0.60) | 0.346 | |
| Compassion | 4.70 (0.76) | 4.65 (0.59) | 0.697 | |
| Positive | 4.81 (0.49) | 4.63 (0.62) | 0.106 | |
| Explaining | 4.70 (0.76) | 4.63 (0.59) | 0.599 | |
| Control | 4.62 (0.72) | 4.57 (0.65) | 0.693 | |
| Plan | 4.68 (0.71) | 4.52 (0.64) | 0.225 | |
| Overall CARE score | 45.76 (7.25) | 43.80 (6.53) | 0.147 | |
| Linear empathy score |  |  |  | |
| Warm | 9.38 (1.05) | 8.91 (1.28) | 0.043 | |
| Pleasant | 9.37 (1.63) | 8.95 (2.07) | 0.254 | |
| Compassionate | 9.18 (1.84) | 8.96 (1.43) | 0.494 | |
| Caring | 9.20 (1.80) | 9.04 (1.61) | 0.614 | |
| Sensitive | 9.39 (1.64) | 9.21 (1.06) | 0.516 | |

CARE item definitions: Ease, making you feel at ease; Story, letting you tell your “story”; Listening, really listening; Interested, being interested in you as a whole person; Understanding, fully understanding your concerns; Compassion, showing care and compassion; Positive, being positive; Explaining, explaining things clearly; Control, helping you to take control; Plan, making a plan of action with you.

Abbreviations: SD, standard deviation; CI, confidence interval; CARE, Consultation and Relational Empathy.
